# Simultaneous Quantification of Five Bioactive Components of *Acanthopanax senticosus* and Its Extract by Ultra Performance Liquid Chromatography with Electrospray Ionization Time-of-Flight Mass Spectrometry

**DOI:** 10.3390/molecules17077903

**Published:** 2012-06-29

**Authors:** Shi-Ping Liu, Jing-Tao An, Rui Wang, Qiang Li

**Affiliations:** 1 Department of Pharmacy, The First Affiliated Hospital of Harbin Medical University, Harbin 150001, China; 2 Department of Orthodontics, School of Stomatology, Harbin Medical University, Harbin 150001, China; 3 Department of Pediatrics, School of Stomatology, Harbin Medical University, Harbin 150001, China; 4 College of Pharmacy, Beijing University of Chinese Medicine, Beijing 100029, China

**Keywords:** *Acanthopanax senticosus*, *Acanthopanax *extract, UPLC-TOF-MS, quantitative analysis

## Abstract

A simple and reliable ultra performance liquid chromatography coupled with electrospray ionization time-of-flight mass spectrometry method (UPLC-TOF-MS) was developed and validated for the simultaneous determination of the major bioactive constituents in *Acanthopanax senticosus* and its extract. The separation of five compounds was performed on a UPLC^TM^ HSS T3 column (100 mm × 2.1 mm, 1.7 μm) with gradient elution using a mobile phase consisting of 0.1% aqueous formic acid and acetonitrile containing 0.1% formic acid. All targeted compounds (syringin, chlorogenid acid, caffeic acid, eleutheroside E and isofraxidin) were baseline separated within 5.3 min in samples, which represented an approximate six-fold reduction in the analysis time in comparison to published HPLC method. Quantitation was carried out working in the V mode using the narrow widow extracted ion chromatograms (nwXICs) of each compound (extracted using a 20 mDa window). Furthermore, all calibration curves showed good linearity (r > 0.999) within the test ranges. The precision was evaluated by intra- and inter-day tests, which revealed relative standard deviation (RSD) values of less than 3.88%. The recoveries for the quantified compounds were between 96.3% and 103.7%, with RSD values below 2.89%. According to the literature, this study represents the first investigation of the simultaneous analysis of multiple components and the method can be applied to determine the amounts of the major compounds in *Acanthopanax senticosus* and its extract by UPLC-TOF-MS.

## 1. Introduction

*Acanthopanax senticosus* (Rupr. Maxim) Harms (Araliaceae) is a well-known Traditional Chinese Medicine in China, which has been officially listed in the Chinese Pharmacopoeia for a long time [1,2]. It possesses various pharmacological effects, such as antibacterial, antifatigue, anti-oxidant and anti-tumor activities [3,4,5]. Recently, Acanthopanax extract was also recorded in the 2010 edition of the Chinese Pharmacopoeia as a new drug [2]. It has also been shown that Acanthopanax extracts can be used as tonics and sedatives for nourishing the liver and kidneys, and strengthening the bones and muscle [6]. *A.senticosus* or its extract have been shown to contain many effective constituents, including syringin, caffeic acid, chlorogenid acid, eleutheroside E and isofraxidin, *etc.* [7]. Syringin and eleutheroside E were considered to possess significant anti-inflammatory effects [8]. Syringin was also proven to be active in protecting against neuritic atrophy and cell death under Abeta treatment [9]. Isofraxidin has demonstrated antifatigue, antistress and immune-accomondating effects [10]. Caffeic acid and chlorogenic acid are the main active lignans of *A.senticosus*, which has obvious anti-oxidant activities [11]. Therefore, the quality control of *A.senticosus* or its extract should be focused on the determination of multiple active compounds. It is not reasonable to adopt a single active constituent to evaluate the quality of *A.senticosus* as one or two constituents could not be responsible for the overall pharmacological activities of *A.senticosus* or its extract. However, only syringin was selected as quantitative constituent for *A.senticosus* in the 2010 edition of the Chinese Pharmacopoeia. At least the five active compounds mentioned above should be measured simultaneously as the marker constituents for quality control of *A.senticosus* and its extracts.

**Figure 1:**
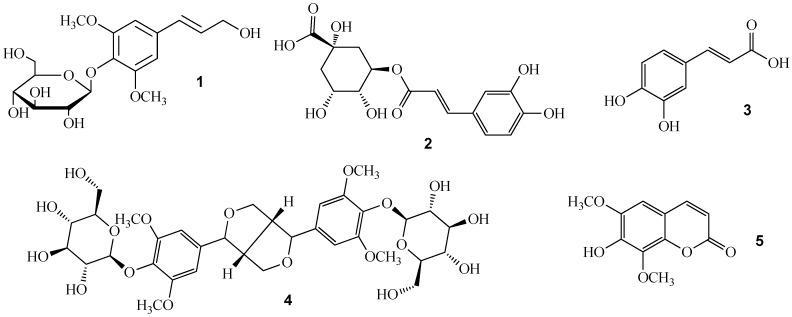
Structures of syringing (**1**), chlorogenic acid (**2**), caffeic acid (**3**), eleutheroside E (**4**) and isofraxidin (**5**).

In the present study, a simple, reliable and new ultra performance liquid chromatography coupled with electrospray ionization time-of-flight mass spectrometry method (UPLC-TOF-MS) was developed and validated for the simultaneous determination of these five major bioactive constituents in *A. senticosus* and its extracts and successfully applied for the assessment of fifteen commercial samples. The structures of compounds **1**–**5** can be seen in Figure 1. According to the literature, this study represents the first investigation of the simultaneous analysis of multiple components in *A. senticosus* and its extract by UPLC-TOF-MS.

## 2. Results and Discussion

### 2.1. Optimization of the Chromatographic Conditions

The selection of mobile phase was a key factor in achieving good chromatographic behavior and appropriate ionization. The resolution of UPLC was tested using both the standard solution and sample solutions. The effect of different mobile phase compositions on chromatographic separation was compared. Acetonitrile-water gave better resolution and peak shapes than methanol-water. Several mobile phase additives, such as formic acid, acetic acid and ammonium acetate were used to achieve better resolution of the analytes. It was found that the good signal intensity, resolution and peak shape were achieved when formic acid was added to both acetonitrile and aqueous solution. But with the increasing added amount of formic acid, the signal intensity showed a downward trend. Ultimately, 0.1% formic acid added to the mobile phase was suitable. 

**Figure 2:**
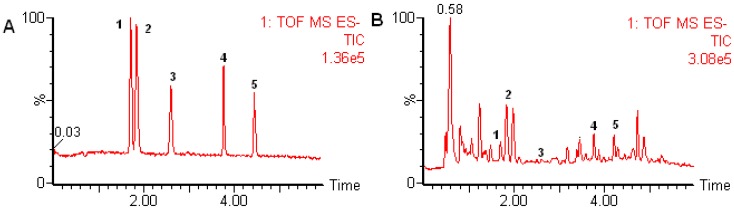
Total ion chromatogram in negative ion mode of standard (**A**) and sample solutions (**B**); **1**: syringing; **2**: chlorogenic acid; **3**: caffeic acid; **4**: eleutheroside E; **5**: isofraxidin.

**Figure 3:**
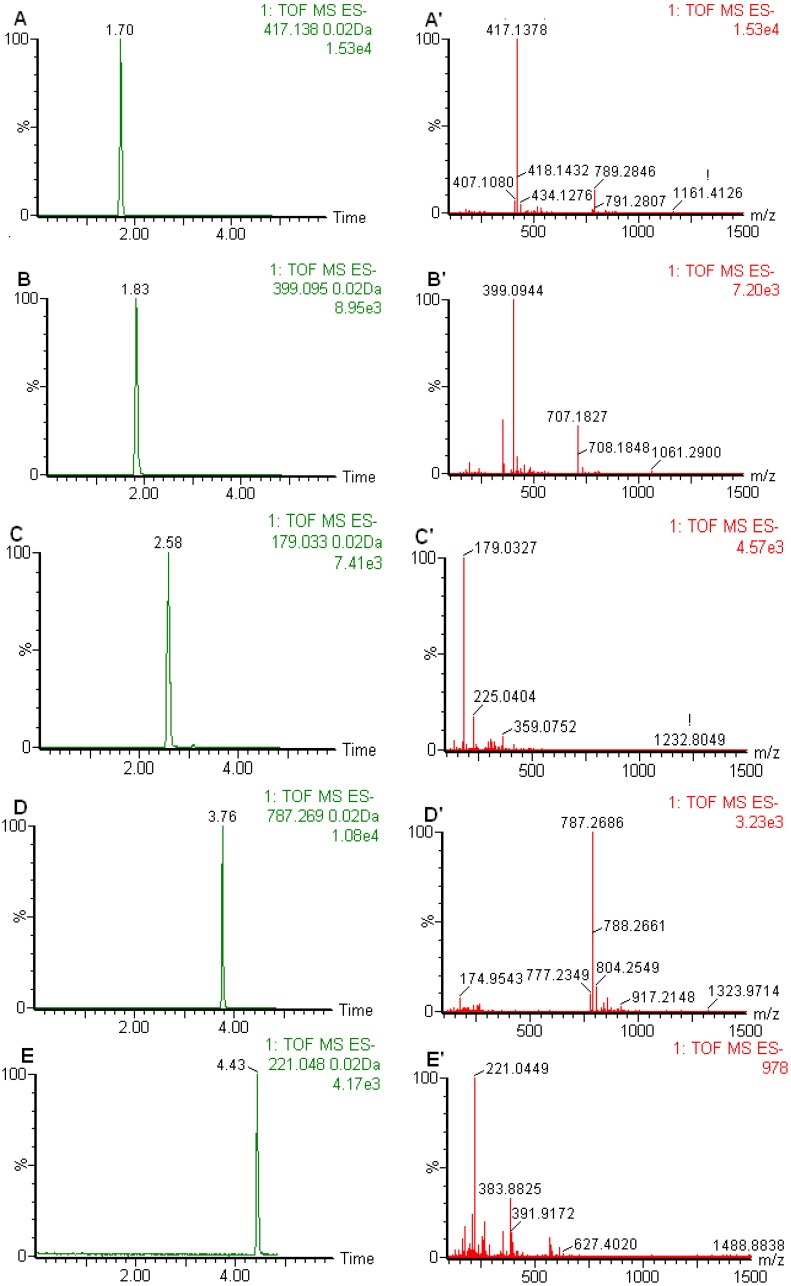
Representative nwXIC chromatograms of standard solutions (A–E) and corresponding mass spectrum (A'–E'). A and A', syringing; B and B', chlorogenic acid; C and C', caffeic acid; D and D', eleutheroside E; E and E', isofraxidin.

The five components were analyzed by MS in ESI negative ion mode. It can be seen from Figure 2 that all targeted compounds syringin (**1**), chlorogenic acid (**2**), caffeic acid (**3**), eleutheroside E (**4**) and isofraxidin (**5**) were baseline separated within 5.3 min in standards and samples, which represented an approximate six-fold reduction in the analysis time in comparison to published HPLC method [7]. Quantitation was carried out working in the V mode using the narrow widow extracted ion chromatograms (nwXICs) of each compound (extracted using a 20 mDa window) (Figure 3A–E). In ESI negative ion of V mode, [M+HCOO]^−^ (417.1378, 399.0949 and 787.2695) for compounds **1**, **2** and **4** and [M−H]^−^ (179.0334 and 221.0482) for compounds **3** and **5** were considered to have better stability and higher abundance (Figure 3A'–E'). Therefore, the narrow widow extracted ions 417.1378, 399.0949, 179.0334, 787.2695 and 221.0482 were chosen for quantitation for compounds **1**–**5**. The analytes were identified by comparing their retention times and accurate *m/z* data with standard solutions containing the corresponding compounds. The typical chromatograms for the standard mixtures analyzed using the optimum chromatographic condition are shown in Figure 2A.

### 2.2. Calibration curves, Limits of Detection and Quantification

Stock solutions were diluted to appropriate concentrations in order to plot calibration curves and calculate relative response factors. The calibration curve of the individual standards was constructed using six concentrations (*n *= 3), by plotting peak areas against the concentration of analytes. The calculated results are shown in Table 1. Good linearity (*r *> 0.999) was observed in calibration curves over the concentration ranges investigated. Limits of detection of six compounds varied from 0.008 μg/mL to 0.20 μg/mL, and limits of quantification ranged from 0.03 to 0.60 μg/mL. The results are summarized in Table 1.

**Table 1:** Calibration parameters of UPLC-MS analysis for the 5 compounds. (**1**: syringin; **2**: chlorogenic acid; **3**: caffeic acid; **4**: eleutheroside E; **5**: isofraxidin).

No.	Regression equation	Linear range (μg/mL)	r	LODs (μg/mL)	LOQs (μg/mL)
1	y = 24.49x + 91.44	0.2–1000	0.9994	0.008	0.03
2	y = 14.70x + 75.46	0.2–1000	0.9992	0.02	0.06
3	y = 14.86x + 78.13	1.0–1000	0.9991	0.04	0.12
4	y = 11.15x + 49.04	0.2–1000	0.9994	0.008	0.03
5	y = 5.49x + 29.14	1.0–1000	0.9991	0.20	0.60

### 2.3. Method Validation

Method precision was checked by intra-day and inter-day variability. The samples (A) were prepared as described in the Experimental part. For the intra-day variability test, the samples were analyzed in six replicates once a day, while for the inter-day variability test, the samples were examined for three consecutive days. The RSD was taken as a measure of precision. From the results obtained, the present method was found to show acceptable precision and accuracy, with intra-day variability RSD values between 0.92% and 3.47% and the inter-day variability RSD values between 1.43% and 3.88%.

A recovery study was performed to validate the accuracy of the developed method. Sample 9 of Acanthopanax extract (0.2 g) was spiked with different concentration levels (50, 100 and 150%) of known amounts of the compounds **1**–**5**. The spiked samples were extracted with 10 mL methanol following the procedure for sample preparation as described above. The recovery was determined by comparing the amount of analyte added to the sample and the amount of analyte detected during UPLC-TOF-MS analysis. The recoveries (Table 2) of the five analytes were in the range of 96.30–103.70%, with RSDs less than 2.89%. The accuracy of the proposed method was therefore found to be sufficient for the determination of the five compounds in samples from *A. senticosus* and its extract.

**Table 2:** Recovery experiment of analytical method for five components. (**1**: syringin; **2**: chlorogenid acid; **3**: caffeic acid; **4**: eleutheroside E; **5**: isofraxidin).

No.	Original (mg)	Spiked (mg)	Found (mg)	Mean recovery (%)	RSD (%) (n = 3)
1	1.022	0.533	1.545	98.13	1.52
		1.066	2.103	101.41	1.78
		1.599	2.563	96.37	2.06
2	0.638	0.262	0.899	99.62	1.05
		0.524	1.176	102.67	2.14
		0.786	1.405	97.58	1.25
3	0.044	0.033	0.078	103.03	0.96
		0.066	0.109	98.48	1.49
		0.099	0.145	102.02	2.82
4	1.172	0.608	1.773	98.85	1.76
		1.216	2.380	99.34	1.03
		1.824	2.994	99.89	2.89
5	0.050	0.027	0.076	96.30	0.89
		0.054	0.106	103.70	1.81
		0.081	0.131	98.77	1.67

### 2.4. Matrix Effects

Evaluation of the matrix effects on the results of quantitative determination of multiple components in TCMs is an important and often overlooked element. Matrix effects occur when matrix compounds co-eluting with the analytes alter the ionization efficiency of the electrospray interface. Post extraction addition is an effective method for providing favorable results, even with variable matrices. The sample of *A. senticosus* was extracted as described. Next, 5 mL of the extract was spiked with a one- fold mixed standard solution at three concentration levels (50%, 100% and 150%), and another 5 mL of the extract was diluted one-fold with methanol. Triplicate samples were prepared at each level. The matrix effect was calculated by the formula: matrix effect (%) = (a − b)/c × 100%, where a is the peak area of the analyte in the spiked sample matrix, b is the peak area of the analyte in the unspiked sample matrix and c is the peak area of the standard solution at the same concentration. No matrix effect is observed when the matrix effect (%) is equal to 100%. In this study, the matrix effects of the eight compounds were in the range of 98.6–101.3%, indicating that no matrix effect was observed.

### 2.5. Sample Analysis

The optimum conditions were applied to the determination of compounds **1**–**5** in *A. senticosus* and its extract from different sources. The typical chromatograms obtained are shown in Figure 2B. The quantitative analyses were performed by means of the external standard methods. The contents of the five compounds in *A. senticosus* and its extract from different sources are listed in Table 3. The quantitative analytical results indicated that their contents showed great variations. The contents of compound **1** ranged from 0.855 mg/g to 13.551 mg/g in samples 1–12 corresponding to Acanthopanax extract and from 0.067 mg/g to 0.58 mg/g in samples 13–15 corresponding to *A. senticosus* raw materials. Similarly, the contents of compounds **2**, **4** and **5** in Acanthopanax extract ranged from 2.035 mg/g, 1.453 mg/g and 0.103 mg/g to 17.638 mg/g, 23.617 mg/g and 1.967 mg/g, respectively. Compound **3** was absent or not detected in samples 1–7, 10, 12 and 13. Although the differences of the content between the samples from different sources were obvious, it is difficult to distinguish the sources of the samples. Generally, the variations were based on internal factors such as genetic variation and plant origin as well as external factors including seasonal, environmental factors, harvest time, and storage conditions and extract process. To ensure the quality of Acanthopanax extract, this suggests that each procedure involved should be standardized. 

**Table 3:** The measurement results of compounds **1**–**5** in *A. senticosus* and its extract (mg/g).

No.	1 (syringin)	2 (chlorogenic acid)	3 (caffeic acid)	4 (eleutheroside E)	5 (isofraxidin)
1	12.857	10.131	Trace	21.004	1.150
2	11.326	11.844	Trace	14.530	1.446
3	13.551	12.146	Trace	16.208	1.586
4	4.364	12.057	Trace	13.853	0.841
5	4.919	13.721	Trace	15.582	1.036
6	9.235	17.638	Trace	23.617	1.967
7	0.906	3.508	Trace	10.108	1.967
8	5.242	4.708	0.308	6.273	0.225
9	5.109	3.191	0.219	5.861	0.248
10	0.855	2.039	Trace	1.453	0.103
11	5.492	2.035	0.262	6.350	0.270
12	0.894	2.637	Trace	1.603	0.107
13	0.067	0.369	Trace	0.424	Trace
14	0.580	1.240	0.041	0.620	0.019
15	0.285	0.648	0.035	0.192	0.026

### 2.6. Quality Assessment of *A. senticosus* Samples by Principal Component Analysis

**Figure 4:**
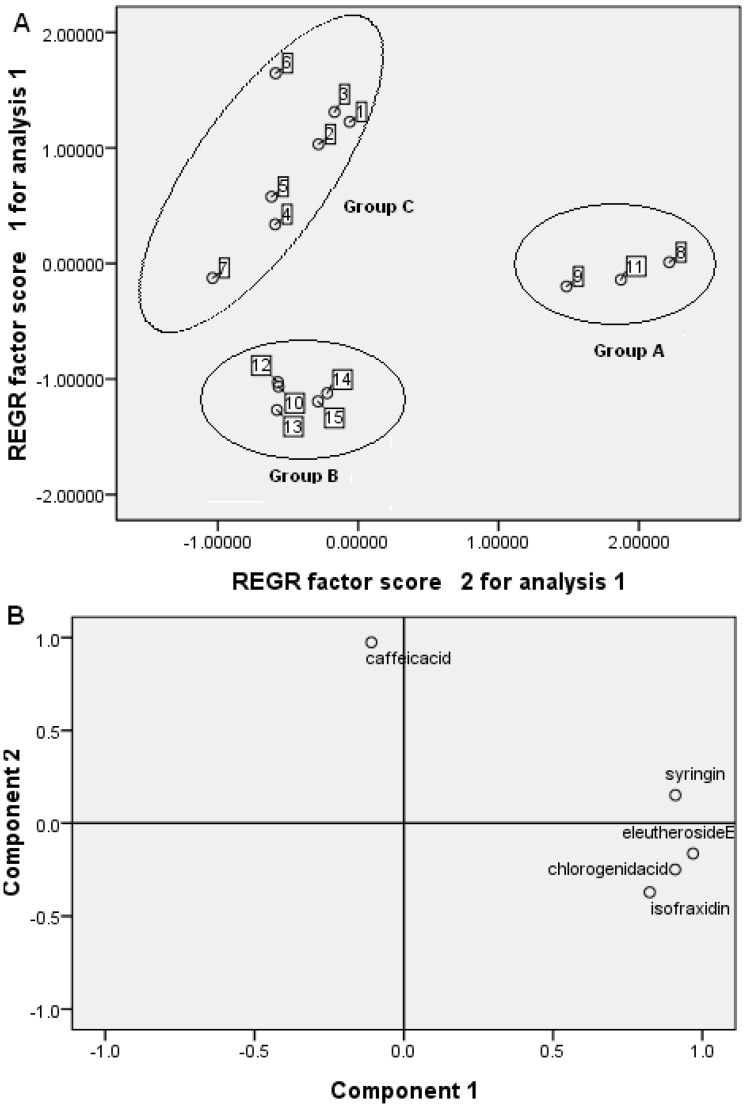
(**A**) Score plots from PCA; (**B**) loading plots from PCA.

The data of these five analytes were used to carry out principal component analysis (PCA). The 5 × 15 auto-scaled data matrix was then submitted to PCA analysis by an SPSS version 16.0 statistical analysis package (SPSS Inc., Chicago, IL, USA). A two-component score plot of UPLC data was utilized to depict general variations of samples among the different sources. The clear separation of different *A. senticosus* and its extract samples was observed in the PCA score plot, where each coordinate represents a sample. The examination of the score plot demonstrated that PCA of UPLC data obtained from 15 samples divided the samples into three groups (Figure 4A). 

Samples 8, 9 and 11 from the factory of Wusulijiang were attributed to group A, which represents raw materials from Shangzhi of Heilongjiang. The samples 1–7 for group B were from two factories of Wusulijiang and Renhuang, which represent raw materials from Wuchang of Heilongjiang. The samples 10 and 12 from the factory of Wusulijiang were similar to raw material samples 13–15, which were attributed to group C. The difference between these *A. senticosus* samples is a result of them coming from different manufacturers, plant origins, harvest time and extract processes. The loading plot was utilized to identify the differential analytes accountable for the separation among different groups (Figure 4B). For example, all targeted compounds showed negative association with group B, which are also in accordance with the fact that the contents of syringin, chlorogenic acid, caffeic acid, eleutheroside E and isofraxidin are much lower in samples 10 and 12–15 than other samples.

## 3. Experimental

### 3.1. Chemicals and Reagents

Chromatography grade acetonitrile was purchased from Merck (Darmstadt, Germany). Deionized water was purified by Milli-Q system (Millipore, Bedford, MA, USA). Detailed information of *A. senticosus* and its extract can be seen in Table 4. Standards of syringin (**1**), chlorogenic acid (**2**), caffeic acid (**3**), eleutheroside E (**4**) and isofraxidin (**5**) were all ordered from the Chinese National Institute of Control of Pharmaceutical and Biological Products (Beijing, China). Their structures can be seen in Figure 1. All other reagents were of analytical grade.

**Table 4:** Detailed information of *A. senticosus* samples.

Samples	Plant origins	Collection time	Factories	Properties
1–3	Wuchang, Heilongjiang	March 2011	Renhuang	Extract
4–7	Wuchang, Heilongjiang	January 2011	Wusulijiang	Extract
8–9	Shangzhi, Heilongjiang	December 2010	Wusulijiang	Extract
10	Harbin, Heilongjiang	April 2011	Wusulijiang	Extract
11	Shangzhi, Heilongjiang	March 2011	Wusulijiang	eExtract
12	Harbin, Heilongjiang	May 2011	Wusulijiang	Extract
13	Shangzhi, Heilongjiang	November 2010	-	Medical materials
14	Wuchang, Heilongjiang	September 2010	-	Medical materials
15	Anhui	February 2011	-	Medical materials

### 3.2. Sample Preparation of Acanthopanax Extract

The 12 batches of Acanthopanax extract were prepared for each pharmaceutical factory by the same method recorded in Chinese Pharmacopoeia 2010. Namely, 1,000 g of *A. senticosus *were ground to a suitable particle size and boiled in water for 3 h each time (×2) and the combined solution was filtered and concentrated under vacuum and the concentrate were finally prepared by spray drying.

### 3.3. Analytical Sample Preparation

The dried powders of Acanthopanax extract (1.0 g) were accurately weighed and dissolved in a 25 mL volumetric flask with methanol. The methanol solution was filtered through a 0.22 μm filter membrane for UPLC injection. The dried powders of *A.senticosus* (1.0 g, 60 mesh) were accurately weighed and extracted with ultrasonic assistance with 10 mL of methanol solution for 30 min. Then the resultant mixture was adjusted to the original weight and the supernatant were filtered through a 0.22 μm membrane before UPLC injection.

### 3.4. UPLC-MS Conditions

The UPLC-MS analysis was performed on a Waters ACQUITY UPLC system (Waters Corporation, Milford, PA, USA) coupled with a Waters LCT Premier XE equipped with electrospray ionization. For the reversed-phase UPLC analysis, the ACQUITY UPLC^TM^ HSS C18 column (100 mm × 2.1 mm i.d., 1.7 μm, Waters Corporation) was used. The column temperature and sample temperature were maintained at 40 °C and 4 °C, respectively; the flow rate of the mobile phase was 0.40 mL/min; the injection volume was fixed at 1.0 μL. Mobile phase A consisted of 0.1% formic acid in acetonitrile, while mobile phase B consisted of 0.1% formic acid in water. The column was eluted with a linear gradient of 12–13.5% A over initial to 2.0 min, 13.5–45% A over 2.0–7.0 min, 45–65% A over 7.0–8.0 min, 65–100% A over 8.0–9.0 min.

The mass spectrometric full-scan data were acquired in the negative ion by V mode from 100 to 1,500 Da with a 0.1 s scan time. Other conditions were as follows: capillary voltage of 1.8 kV, desolvation temperature of 350 °C, sample cone voltage of 60 V, extraction cone voltage of 4.0 V, source temperature of 110 °C, cone gas flow of 20 L/h and desolvation gas flow of 750 L/h for negative ion mode. Data were centroided and mass was corrected during acquisition using an external reference (Lock-Spray^TM^) consisting of a 0.2 ng/mL solution of leucine enkephalin infused at a flow rate of 50 μL·min^−1^ via a lockspray interface, generating a reference ion for negative ion mode ([M−H]^−^ = 554.2615) to ensure accuracy during the MS analysis.

### 3.5. Standard Preparation and Calibration Curves

A methanol stock solution containing all five reference standards was prepared by dissolving the reference standards in methanol to final concentration of 1,000 μg/mL for each reference standard, then diluted the mixture stock solution to an appropriate concentration to establish calibration curves. Each calibration curve concentration was done in triplicate. All calibration curves were constructed from peak areas of reference standards *versus* their concentrations. The lowest concentration of working solution was diluted with methanol to yield a series of appropriate concentrations, and the LOD and LOQ under the chromatographic conditions were separately determined at an S/N of 3 and 10, respectively. The results are summarized in Table 1. 

## 4. Conclusions

A new UPLC-TOF-MS method has been developed for the first time for the simultaneous determination of five major active constituents in Acanthopanax extract and raw materials. The validation data indicated that this method is reliable and can be applied to determine the contents of these five compounds in Acanthopanax extract and raw materials from different sources. This valuable information concerning the concentration of these pharmacologically active constituents in Acanthopanax extract and raw materials could be of great importance for the quality assessment and should therefore be useful for the guidance of clinical use. This new assay also meets the need for simultaneous determination of multiple constituents in Acanthopanax extract and raw materials by UPLC-TOF-MS, which reduced the sample handling and analytical time by six-fold, and the detection limit by five- to 1,000-fold, compared to published methods [7].
